# Sex differences in pediatric major depressive episodes: a cross-sectional study on psychiatric symptoms in early-onset mood disorders

**DOI:** 10.3389/fpsyt.2025.1503794

**Published:** 2025-04-25

**Authors:** Massimo Apicella, Elisa Andracchio, Giorgia Della Santa, Caterina Lanza, Clotilde Guidetti, Monia Trasolini, Maria Elena Iannoni, Gino Maglio, Stefano Vicari, Giulia Serra

**Affiliations:** ^1^ Child and Adolescent Neuropsychiatry Unit, Bambino Gesù Children’s Hospital, Istituto di Ricovero e Cura a Carattere Scientifico (IRCCS), Rome, Italy; ^2^ Department of Neuroscience, Università Cattolica del Sacro Cuore, Rome, Italy; ^3^ Department of Psychiatry, Massachusetts General Hospital, Harvard Medical School, Boston, MA, United States; ^4^ Department of Life Sciences and Public Health, Università Cattolica del Sacro Cuore, Rome, Italy

**Keywords:** depression, bipolar, adolescent, sex, gender, comorbidities, neurodevelopment, psychopathology

## Abstract

**Background:**

Sex differences in psychiatric symptoms among children and adolescents with a major depressive episode (MDE) are less studied than among adults. Previous non-recent studies reported a greater severity in adolescent girls and small differences between sexes in specific symptoms. We aim to explore the differences between male and female patients in the diagnoses, comorbidities, and psychiatric symptoms in a large cohort of pediatric patients referred to a tertiary center for child and adolescent psychiatry.

**Methods:**

We collected cross-sectional data on 382 consecutively referred patients (age 6–18 years; 73.8% female patients) with current MDEs (unipolar or bipolar) thoroughly evaluated with clinician (Children’s Depression Rating Scale-Revised; K-SADS Mania Rating Scale; Columbia Suicide Severity Rating Scale) and self and parent report (Children’s Depression Inventory-2; Multidimensional Anxiety Scale for Children-2; Child Behavior Checklist) standardized measures. Bivariate analyses were followed by a logistic regression model to assess significant predictors of the MDE phenotype of female (vs. male) patients.

**Results:**

Female patients were more likely to show severe MDEs (41.5% vs. 26.0%; *p* = 0.006), suicidal ideation (63.9% vs. 47.0%; *p* < 0.001) and behaviors (29.4% vs. 13.0%; *p* = 0.001), and non-suicidal self-injury (58.5% vs. 27.0%; *p* < 0.001). Male patients were more frequently diagnosed with bipolar disorder (21% vs. 11%; *p* = 0.012) and/or comorbid ADHD/behavior disorders (20% vs. 8.9%; *p* = 0.003). Male patients also had more frequently significant mixed hypo/manic symptoms (17% vs. 7.7%; *p* = 0.01) and were younger at the onset of the first psychiatric symptom (6.32 vs. 7.75 years; *p* = 0.003), onset of mood disorder (11.3 vs. 12.5 years; *p* = 0.005), and evaluation (14.0 vs. 15.2 years; *p* = 0.001). Several symptoms were significantly and independently associated with female patients diagnosed with a current MDE, including a) excessive weeping (OR 1.53; *p* < 0.001), b) mood lability (OR 1.50; *p* = 0.014), c) excessive fatigue (OR 1.38; *p* = 0.002), d) appetite disturbance (OR 1.28; *p* = 0.041), and e) attention problems (OR 1.07; *p* = 0.001). Distractibility (OR 0.55; *p* = 0.009) and conduct problems (OR 0.93; *p* = 0.001) were in turn correlated with MDE among male patients.

**Discussion:**

The study confirms that female patients with MDEs exhibit more severe affective symptoms, while male patients present with more externalizing behaviors and comorbidities. We further report more mixed symptoms and bipolar disorder diagnoses in male patients, who also have an earlier onset of psychiatric symptoms. These findings are discussed also considering implications for the diagnosis of pediatric bipolar disorder. A high clinical sensitivity is needed for highlighting subtle mixed and/or atypical features in severe MDEs among girls.

## 1 Introduction

Depression is a prevalent psychiatric disorder globally and one of the leading causes of both mental and physical disability worldwide according to the World Health Organization (1). The pooled prevalence rate of depressive disorders in children and adolescents in Europe is 1.7% (2). Its burden is even greater in lower-middle-income countries (3). The prevalence of depression before puberty is similar in both sexes (4), and boys and girls diagnosed with major depressive disorder (MDD) exhibit comparable symptoms before puberty (5). During adolescence, the prevalence of depression among female patients doubles, resulting in a 1:2 male-to-female ratio in both adolescents and adults (4). Studies on adult samples report sex differences in depressive symptoms, with more externalizing behaviors among men and more severe depressive symptoms among women (6). Indeed, men are reported to display more likely externalizing behaviors, impulsivity, and risk-taking, which are not included in the DSM-5 criteria for major depressive episode (MDE) and may serve as maladaptive coping strategies (6, 7). Women are more likely to experience more severe depressive symptoms (8), report them more openly, and present with greater appetite disturbances (9). They are also more likely to have comorbid internalizing disorders, such as anxiety and eating disorders (6). The effect sizes of differences in specific symptoms remain small (≤0.2) (6).

Research on sex differences in adolescent depression is less extensive than in adults. In general population samples, female patients have been reported to express higher self-rated depressive symptoms compared to male patients, findings that most studies contextualized within the complex biopsychosocial interactions during adolescent development. Emotional reactivity to interpersonal stressors has been reported to be higher among girls (10), and specific stressors, including the absence of social support, may impact sexes differently (11). According to a theoretical model, female patients have increased affiliative needs during puberty, which makes them more susceptible to interpersonal stressors as a risk factor for the development of depressive symptoms (12).

The literature on adolescents with a confirmed clinical diagnosis of depression is more limited and has shown mixed results. Some authors found no clear gender differences in depressive features in clinically referred adolescents (13, 14); however, other studies showed that female adolescents with depression were more likely to report sadness, depressed mood, self-disappointment, self-blame, feelings of failure, concentration difficulties, fatigue, and health worries, while male adolescents were more likely to report anhedonia and worsened mood in the morning (15) and to display more impulsive and antisocial behaviors (16). Coping strategies may also differ: female adolescents tend to ruminate, while male adolescents more often avoid emotional exploration (17). Female adolescents with depression exhibit greater self-doubt, self-blame, and negative self-evaluation, with a need for external validation (16).

Cultural differences may have an impact on the expression of psychiatric symptoms (18) and may be relevant in interpreting reports on gender differences in depression; however, studies from different cultural contexts report consistent findings. In a sample of adolescents with confirmed DSM-IV MDD from China, girls were found to experience higher levels of depression and anxiety symptoms (19). In a sample of Japanese adolescents, higher emotional expressivity and lower self-efficacy, competitiveness, and risk-taking behaviors were described in female adolescents (20).

Epidemiological studies indicate a higher lifetime incidence (149 vs. 55.3 per 100,000/year) (21) and prevalence (3.3% vs. 2.6%) (22) of bipolar disorder (BD) in female adolescents. Male adolescents more frequently meet the criteria for hypo/manic episodes, while female adolescents report more depressive symptoms, particularly at older ages (23). In a previous study by our group, women with MDEs and mixed features exhibited greater affective symptoms, fatigue, and emotional dysregulation, while men showed more psychomotor activation (24). This remains the only study to date assessing gender differences in mood disorders using measures for both depressive and hypo/manic symptoms, emotional dysregulation, and problematic behaviors.

Research on sex differences in adolescent depression—especially in clinical populations assessed with clinician-rated and multi-informant scales—remains limited, but some evidence suggests that sex-specific factors significantly shape symptoms, with implications for neurobiology and social influences on early-onset mood disorders. Additionally, mixed hypomanic symptoms, often overlooked in standard depression assessments, are common also in children and adolescents with severe early-onset mood disorders and are crucial for evaluating clinical severity (25). Our study examines sex differences in mood disorder diagnoses and psychiatric symptoms in a large clinical population of children and adolescents over nearly a decade, incorporating clinician, parent, and self-report measures.

## 2 Methods

### 2.1 Participants

The sample for this study was recruited from a day hospital unit of a specialized psychiatric service for the assessment and treatment of early-onset mood disorders, managed by the Mood Disorders Program team at Bambino Gesù Children’s Hospital in Rome. Subjects were referred to confirm a diagnostic hypothesis of a moderate/severe mood disorder. Referral sources included primary care doctors (pediatricians and general practitioners), secondary care providers (child psychiatrists in national healthcare centers), and the emergency department of Bambino Gesù Children’s Hospital.

Historical data obtained during clinical assessments performed in our outpatient service from June 2013 to March 2022 were extracted from clinical records. We included consecutively referred children and adolescents, aged 6 to 18 years at the time of evaluation. Categorical diagnoses were clinically made and confirmed with the Kiddie-Schedule for Affective Disorders and Schizophrenia for School-aged Children, Present and Lifetime version (K-SADS-PL) (26) following the DSM-5 criteria. We included subjects diagnosed with a current MDE in the context of either an MDD or BD. Subjects diagnosed with persistent depressive disorder with intermittent major depressive episodes, with current MDE, and subjects diagnosed with MDE with mixed symptoms were also included.

We excluded patients with MDE in partial or complete remission at the time of evaluation, those with intellectual disability, autism spectrum disorder, substance-induced mood disorders, or mood disorders due to other medical conditions, as defined by DSM-5. Parents/legal guardians provided written informed consent for anonymous reports of data in aggregate form, in compliance with research ethics. Data collected were stored in individual clinical records. The study has been conducted according to the guidelines established in the Declaration of Helsinki. Considering the retrospective nature of the analysis, the current study did not require the approval of the local ethics committee according to current legislation, but a notification was sent (Comitato Etico Ospedale Pediatrico Bambino Gesù practice number 3380). Data were retrospectively anonymously analyzed in line with personal data protection policies.

### 2.2 Assessment

All subjects were evaluated over a minimum of three appointments, totaling 9–10 h of clinical assessment. All study participants were assessed by an experienced child and adolescent psychiatrist and an experienced psychologist using the K-SADS-PL (26), a semistructured, clinician-administered diagnostic interview performed with both subjects and their parents or adult legal representatives to assess current and past psychopathological features and psychiatric disorders, including primary mood disorders and comorbidities in juveniles, according to the DSM-5 criteria. Childhood clinical features (syndromal or subsyndromal) preceding the onset of current disorders were systematically assessed and coded both as “traits” if subsyndromal or “disorder” if meeting the criteria for a DSM-5 diagnosis, with particular attention to the time of onset of first mood and non-mood disorder-related psychiatric symptoms (27).

Additionally, depressive and hypo/manic or mixed symptoms were rated by the same experienced clinicians using the Children’s Depression Rating Scale-Revised (CDRS-R) (28) and the K-SADS Mania Rating Scale (KMRS) (29). The CDRS-R is a semistructured interview used to rate depressive symptoms in subjects aged 6–18 years, based on 17 items with a raw score range of 17–120 (sum of the scores of single items, considered positive at scores >30 corresponding to a *T*-score >55). We analyzed the CDRS-R total score and the single items’ scores (school dysfunctions, difficulties in having fun, difficulties in interpersonal relationships, sleep disorders, appetite disorders, excessive fatigue, psychosomatic complaints, irritability, excessive guilt, low self-esteem, depressive feelings, morbid ideas, suicidal ideation, excessive crying, reduced facial expressions, slow speech, and motor hypoactivity). The KMRS is a structured interview used to rate hypo/manic symptoms in subjects aged 6–18 years, based on 14 items, with a total score range of 1–68 (sum of the scores of 0 to 6 for single items minus 13, considered indicative of significant hypomanic symptoms at scores >12). We analyzed both the total score and the single items’ score (euphoria and expansiveness; irritability and anger; mood lability; reduced need for sleep; crowded thoughts; increased energy; increased activities; motor hyperactivity; grandiosity; rapid or pressured speech; distractibility; impaired judgment; hallucinations; and delusions). Single items’ score of <2 is considered normal, a score of 2–3 is considered borderline, and a score >3 is considered clinically significant (29). Depression severity at the time of evaluation—whether mild, moderate, severe, with or without psychotic symptoms, or in partial/complete remission—was defined based on clinical judgment according to the DSM-5 criteria and CDRS-R cutoffs: a total score of 30 to 44 for mild, 45 to 55 for moderate, 55 or more for severe depression, and 29 or less for remission.

Suicidal ideation and suicidal behaviors were evaluated with the Columbia Suicide Severity Rating Scale (C-SSRS) (30). Suicidal behavior is defined as any self-harming behavior resulting in any kind of damage with non-zero intent to die, declared by the patient or evident from documented circumstances. Non-suicidal self-injury (NSSI) was systematically investigated lifetime and accurately differentiated from suicidal behavior and defined as in section III of DSM-5 (31).

To assess self-reported symptoms, the Children’s Depression Inventory-2 (CDI-2) (32) and the Multidimensional Anxiety Scale for Children-2 (MASC-2) (33) were administered to patients and their parents/caregivers. For each module of CDI-2 and MASC-2, the total *T*-score was analyzed.

Finally, the Italian version of the Child Behavior Checklist (CBCL) (34) was administered to parents/caregivers to score different behavioral and emotional problems severity. It has strong psychometric properties and is widely used in Italy (35, 36). It provides scores on three behavior rating scales that address internalizing symptoms, externalizing symptoms, and total behavioral problems. Sub-items of these scales include syndromic and DSM-oriented scales.

An overall Deficient Emotional Self-Regulation CBCL profile was calculated by summing scores for attention problems, aggression, and anxious-depressed syndromic scales. This score is reported to be indicative of Deficient Emotional Self-Regulation at scores between 180 and 210 (1 to 2 standard deviations) and as meeting the criteria for a Dysregulation Profile at a >210 (>2 standard deviations) score (37).

### 2.3 Statistical analysis

Categorical variables were presented as counts and frequency rates, while continuous variables were expressed as means and standard deviations. For scales with missing data, participants were excluded from analyses, and no imputation methods were applied.

Associations between unpaired categorical variables were tested using Pearson’s chi-square test, with Fisher’s exact test applied when more than 20% of cells in the contingency table had expected frequencies <5 (38). Mean differences of continuous variables between the two groups were tested using Student’s *t*-test for unpaired data.

We performed a logistic regression analysis with female sex as the dependent variable, to assess which of the studied variables best predict the MDE phenotype in female patients, while controlling for other factors. In more detail, our aim was to determine how accurately and independently the most relevant clinician-, self-, and parent-rated measures can distinguish between male and female patients with a current MDE based on psychiatric symptom expression. Of the CDRS-R items, KMRS items, CBCL scales, and MASC-2 and CDI-2 *T*-scores, variables found significantly different between sexes at previous bivariate analyses were tested as predictors, while female sex was set as the predicted value. Predictor selection was performed using a stepwise backward approach, progressively removing variables with *p >*0.05. Odds ratios (OR), with their 95% confidence intervals (95% CI), *p*-values, and Wald test statistics have been presented. All tests were two-tailed. A *p*-value of 0.05 or less was considered indicative of significance. Analyses were conducted with Microsoft Office 365—Excel and IBM SPSS Statistics V26 software.

## 3 Results

### 3.1 Participants’ demographics and psychiatric diagnoses

After the application of the exclusion criteria, we retrospectively identified 382 patients diagnosed with current MDE who were eligible for inclusion in the study. Of these participants, 73.8% (*N* = 282) were female patients and 26.2% (*N* = 100) were male patients. Male patients were significantly younger at the time of evaluation (14.0 years vs. 15.2 years; *p* = 0.001). They were also younger when their first psychiatric symptom was reported by parents/caregivers (6.32 years vs. 7.75 years; *p* = 0.003) and when the first symptoms of their mood disorder were reported (11.3 years vs. 12.5 years; *p* = 0.005).

Bipolar disorder was diagnosed in 13.6% (*N* = 52) of the participants. This diagnosis was more common in male participants (21% vs. 11%; *p* = 0.012). Conversely, MDD diagnosis was significantly more frequent among female participants (78% vs. 61%; *p* < 0.001).

Thirty-seven percent of the subjects had a severe MDE, with a CDRS-R total score >55. Most patients were classified as having a moderate severity depressive episode (CDRS-R total score between 45 and 55). Severe MDEs were more frequently diagnosed in female than in male participants (41.5% vs. 26.0%; *p* = 0.006); mild MDEs, in turn, were more frequently diagnosed in male participants (33.0% vs. 16.7%; *p* < 0.001).

The most frequent comorbidities were anxiety disorders and obsessive-compulsive or tic disorders (27%), followed by ADHD/behavior disorders (11.8%), learning disorders (11.3%), substance abuse (8.4%), and eating disorders (6.5%). Only ADHD/behavior disorders were significantly more prevalent among male participants than female participants (20% vs. 8.9%; *p* = 0.003). No other significant differences in comorbidities were observed between male and female participants. For further details on participants’ demographics and psychiatric diagnoses, see **Table 1**.

**Table 1:** Descriptive characteristics of the study patients.

Measure	All subjects	Males	Females	*p*-value (statistic)
Subjects (*n*)	382	100	282	–
Age (years, mean ± SD)				
At evaluation At first symptom of mood disorder At first psychiatric symptom	14.9 ± 2.21 12.2 ± 2.87 7.39 ± 3.92	14.0 ± 2.87 11.3 ± 3.53 6.32 ± 3.52	15.2 ± 1.84 12.5 ± 2.54 7.75 ± 3.99	0.001 (3.83) 0.005 (2.89) 0.003 (2.97)
Diagnosis (%, n)				
Bipolar disorders *(I, II, or not otherwise* sp*ecified)* Unipolar depressive disorders Major depressive disorder Persistent depressive disorder	52 330 73.6% (281) 12.8% (49)	21% (21) 79.0% (79) 61.0% (61) 18.0% (18)	11% (31) 89.0% (251) 78.0% (220) 11.0% (31)	0.012 (6.29) 0.012 (6.29) <0.001 (11.0) 0.072 (3.24)
Severity of current MDE (%, n)				
Mild (CDRS-R < 45) Moderate (CDRS-R 45–55) Severe (CDRS-R > 55)	20.9% (80) 41.6% (159) 37.4% (143)	33.0% (33) 41.0% (41) 26.0% (26)	16.7% (47) 41.8% (118) 41.5% (117)	<0.001 (11.9) 0.883 (0.020) 0.006 (7.56)
Psychiatric co-morbidities (%, n)				
ADHD or behavior disorders Anxiety or OCD/tic disorders Eating disorders Learning disorder Substance use (current or lifetime)	11.8% (45) 27.0% (105) 6.50% (25) 11.3% (43) 8.40% (32)	20.0% (20) 26.0% (26) 10.0% (10) 16.0% (16) 11.0% (11)	8.90% (25) 28.0% (79) 5.30% (15) 9.60% (27) 7.40% (21)	0.003 (8.81) 0.698 (0.15) 0.104 (2.65) 0.081 (3.05) 0.270 (1.21)
Self-harm status				
C-SSRS screening score (mean ± SD) Suicidal ideation ever (%, *n*) One or more suicide attempt lifetime (%, *n*) NSSI, current or lifetime (%, *n*)	1.99 ± 1.73 63.9% (244) 25.1% (96) 50.3% (192)	1.02 ± 1.36 47.0% (47) 13.0% (13) 27.0% (27)	2.24 ± 1.73 69.9% (197) 29.4% (83) 58.5% (165)	<0.001 (4.73) <0.001 (16.7) 0.001 (10.6) <0.001 (29.3)
Clinician-rated scales				
CDRS-R (mean ± SD) KMRS (mean ± SD) KMRS score above threshold (%, *n*)	50.8 ± 12.6 6.95 ± 3.91 10.1% (37)	47.2 ± 12.1 7.31 ± 5.27 17.0% (16)	52.0 ± 12.5 6.83 ± 3.32 7.70% (21)	0.001 (3.35) 0.407 (0.833) 0.010 (6.59)
Self-rated scales (mean ± SD)				
MASC-2 T-score CDI-2 T-score	66.7 ± 13.6 73.1 ± 12.6	56.4 ± 12.2 66.1 ± 14.1	69.3 ± 12.8 74.8 ± 11.6	<0.0001 (5.51) 0.001 (3.50)
Parent report scales (mean ± SD)				
MASC-2 T-score CDI-2 T-score CBCL deficient emotion self-regulation profile	68.0 ± 13.5 67.4 ± 9.49 201 ± 21.7	63.2 ± 11.7 68.6 ± 8.77 197 ± 21.8	69.0 ± 13.7 67.1 ± 9.66 202 ± 21.6	0.025 (2.26) 0.413 (0.821) 0.062 (1.88)

*Statistics*: Unpaired *t*-test for continuous and χ2 for categorical measures.

SD, standard deviation; MDE, major depressive episode; CDRS-R, Children’s Depression Rating Scale-revised; ADHD, attention deficit hyperactivity disorder; OCD, obsessive-compulsive disorder; C-SSRS, Columbia-Suicide Severity Rating Scale; NSSI, non-suicidal self-injury; KMRS, Kiddie-SADS Mania Rating Scale; MASC, Multidimensional Anxiety Scale for Children; CDI, Children Depression Inventory; CBCL, Child Behavior Checklist; Deficient Emotional Self-Regulation profile = attention problems + aggression + anxious-depressed index.

### 3.2 Sex differences in suicidal ideation and behaviors

More than 60% of the participants reported having experienced at least one episode of active suicidal ideation during their lifetime. History of active suicidal ideation was more commonly reported by female than male participants (63.9% of female patients vs. 47.0% of male patients; *p* < 0.001). Almost one-third of female subjects reported a lifetime history of one or more suicide attempts, while male subjects reported suicide attempts significantly less frequently (29.4% vs. 13.0%; *p* = 0.001). Consistently, female patients scored higher on the C-SSRS screening version (2.24 ± 1.73 vs. 1.02 ± 1.36; *t* = 4.73; *p* < 0.001). Non-suicidal self-injury (current or lifetime) was also reported in approximately half of the sample and was more commonly reported by female patients (58.5% vs. 27.0%; *t* = 29.3; *p* < 0.001). For further details, see **Table 1**.

### 3.3 Sex differences in clinician-rated symptoms

Depression severity, measured by the CDRS-R total score, was significantly higher among female than male patients (52.0 ± 12.5 vs. 47.2 ± 12.1; *t* = 3.35; *p* < 0.001; **Table 2**). Several depressive symptoms were also significantly more severe in female participants compared to male participants, including excessive weeping (3.51 vs. 2.14; *p* < 0.001), excessive fatigue (3.85 vs. 2.89; *p* < 0.001), low self-esteem (4.37 vs. 3.61; *p* < 0.001), depressive feelings (4.43 vs. 3.67; *p* < 0.001), suicidal ideation (2.67 vs. 2.11; *p* = 0.005), appetite disturbance (2.78 vs. 2.18; *p* < 0.001), physical complaints (3.02 vs. 2.39; *p* = 0.002), and excessive guilt (3.09 vs. 2.41; *p* < 0.001; **Table 2**). No depressive symptom was found to be more severe in male participants.

**Table 2:** Clinician- and parent-rated measures.

Measure	All subjects	Males	Females	*P*-value (unpaired *t*)
Hypo/manic symptoms (KMRS)				
Lability	2.55 ± 0.97	2.35 ± 1.09	2.62 ± 0.92	0.037 (−2.11)
Increased motor activity	1.30 ± 0.62	1.43 ± 0.72	1.25 ± 0.57	0.031 (2.18)
Distractibility	1.42 ± 0.67	1.55 ± 0.79	1.37 ± 0.62	0.047 (2.00)
KMRS total score	6.95 ± 3.91	7.31 ± 5.27	6.83 ± 3.32	0.407 (0.833)
Depressive symptoms (CDRS-R)				
Excessive weeping	3.15 ± 1.79	2.14 ± 1.38	3.51 ± 1.78	<0.0001 (−7.77)
Excessive fatigue	3.60 ± 1.62	2.89 ± 1.66	3.85 ± 1.52	<0.0001 (−5.25)
Low self-esteem	4.17 ± 1.61	3.61 ± 1.52	4.37 ± 1.59	<0.0001 (−4.07)
Depressive feeling	4.23 ± 1.54	3.67 ± 1.47	4.43 ± 1.52	<0.0001 (−4.27)
Suicidal ideation	2.53 ± 1.69	2.11 ± 1.56	2.67 ± 1.72	0.005 (−2.84)
Appetite disturbance	2.62 ± 1.37	2.18 ± 1.39	2.78 ± 1.33	<0.0001 (−3.74)
Physical complaints	2.86 ± 1.71	2.39 ± 1.66	3.02 ± 1.70	0.002 (−3.19)
Excessive guilt	2.91 ± 1.57	2.41 ± 1.50	3.09 ± 1.55	<0.0001 (−3.75)
CDRS-R total score	50.8 ± 12.6	47.2 ± 12.1	52.0 ± 12.5	<0.0001 (−3.35)
Parent-rated symptoms (CBCL 6-18)				
Anxious/depression	72.7 ± 11.0	70.5 ± 10.5	73.5 ± 11.0	0.026 (−2.24)
Somatic complaints	68.2 ± 9.19	66.0 ± 8.99	69.9 ± 9.15	0.011 (−2.57)
Rule breaking behaviors	60.6 ± 8.37	62.6 ± 9.11	59.9 ± 7.89	0.012 (2.53)
Post-traumatic stress problems	72.7 ± 10.2	70.0 ± 9.36	73.7 ± 10.3	0.004 (−2.91)
Attention problems	64.4 ± 9.04	62.6 ± 8.55	65.1 ± 9.13	0.025 (−2.25)
Somatic problems	64.8 ± 9.58	61.9 ± 8.11	65.9 ± 9.89	0.001 (−3.29)
Obsessive-compulsive problems	67.3 ± 10.2	64.8 ± 10.4	68.2 ± 9.93	0.007 (−2.70)
Affective problems	75.8 ± 8.96	73.6 ± 9.18	76.6 ± 8.75	0.007 (−2.70)
Internalizing problems	70.9 ± 9.04	68.3 ± 9.30	71.8 ± 8.76	0.002 (−3.12)
CBCL deficient emotional self-regulation profile	201 ± 21.7	197 ± 21.8	202 ± 21.6	0.062 (−1.88)

*Statistics*: Unpaired *t*-test for continuous variables.

CDRS-R, Children’s Depression Rating Scale-revised; KMRS, Kiddie-SADS Mania Rating Scale; CBCL, Child Behavior Checklist; Deficient Emotional Self-Regulation profile = attention problems + aggression + anxious-depressed index.

The severity of hypo/manic symptoms, measured by the KMRS total score, was similar in mean values between male and female patients, but significant mixed hypo/manic symptoms (defined by the KMRS total score above the instrument threshold of 12) were identified in more male than in female participants (17% vs. 7.7%; *p* = 0.010; **Table 1**). Male patients scored higher than female patients in KMRS increased motor activity (1.43 vs. 1.25; *p* = 0.031) and KMRS distractibility (1.55 vs. 1.37; *p* = 0.047), whereas KMRS mood lability was significantly higher in female patients (2.62 vs. 2.35; *p* = 0.037). For further details on clinician-rated symptoms, see **Table 2**.

### 3.4 Sex differences in self-/parent-rated symptoms

Both self-reported and parent-reported anxiety symptoms, measured by the MASC-2 total score, were significantly higher among female than male patients (69.3 vs. 56.4, *p* < 0.0001 and 69.0 vs. 63.2, *p* = 0.025, respectively). Self-reported depressive symptoms, measured by the CDI-2 total score, were also significantly higher among female than male patients (74.8 vs. 66.1; *p* = 0.001; **Table 1**). Parent reports of depressive symptoms, however, did not show significant differences between groups.

Different internalizing and externalizing parent-rated symptoms and behaviors assessed by the CBCL were different between the sexes. Post-traumatic stress problems (73.7 vs. 70.0; *p* = 0.004), attention problems (65.1 vs. 62.6; *p* = 0.025), internalizing problems (71.8 vs. 68.3; *p* = 0.002), the anxious/depression scale (73.5 vs. 70.5; *p* = 0.026), the somatic complaints scale (69.9 vs. 66.0; *p* = 0.011), obsessive-compulsive problems (68.2 vs. 64.8; *p* = 0.007), and affective problems (76.6 vs. 73.6; *p* = 0.007) were more severe in female than male patients. Conversely, male participants had significantly higher scores in rule-breaking behaviors compared to female participants (62.6 vs. 59.9; *p* = 0.012). For further details on self/parent-rated symptoms, see **Table 2**.

### 3.5 Logistic regression model

To determine the relative importance of each symptom in differentiating between male and female participants with an MDE, a logistic regression model was developed using items that showed significant differences between sexes in the bivariate analyses. The model accurately predicted 80% of cases, correctly classifying 92.5% of female participants and 46.9% of male participants.

The following factors were significantly and independently associated with female gender: a) CDRS-R excessive weeping item score (OR 1.53; *p* < 0.001), b) KMRS lability item score (OR 1.50; *p* = 0.014), c) CDRS-R excessive fatigue item score (OR 1.38; *p* = 0.002), d) CDRS-R appetite disturbance item score (OR 1.28; *p* = 0.041), and e) CBCL attention problems scale score (OR 1.07; *p* = 0.001). Factors significantly and independently associated with male gender included a) KMRS distractibility item score (OR 0.55; *p* = 0.009) and b) CBCL conduct problems scale score (OR 0.93; *p* = 0.001). For further details on the regression model, see **Table 3**.

**Table 3:** The logistic regression model.

Measure	*B* coefficients (standard error)	Odds ratio (95% confidence interval)	Significance (Wald test)
KMRS Lability item	0.407 (0.165)	1.50 (1.09–2.08)	0.014 (6.06)
KMRS Distractibility item	−0.598 (0.228)	0.550 (0.351–0.860)	0.009 (6.88)
CDRS-R Appetite disturbance item	0.246 (0.120)	1.28 (1.01–1.62)	0.041 (4.19)
CDRS-R Excessive weeping item	0.423 (0.106)	1.53 (1.24–1.88)	<0.0001 (15.8)
CDRS-R Excessive fatigue item	0.318 (0.101)	1.38 (1.28–1.68)	0.002 (9.92)
CBCL Attention problems scale	0.064 (0.020)	1.07 (1.03–1.11)	0.001 (10.1)
CBCL Conduct problems scale	−0.068 (0.021)	0.934 (0.897–0.974)	0.001 (10.4)

*Statistics*: Logistic regression model. Predicted variable: female sex.

CDRS-R, Children’s Depression Rating Scale-revised; KMRS, Kiddie-SADS Mania Rating Scale; CBCL, Child Behavior Checklist.

## 4 Discussion

We identified significant sex differences in psychopathology in a large cohort of pediatric patients with current MDEs. Female patients exhibited higher clinician- and self-rated depressive symptoms, while male patients showed a greater prevalence of mixed hypomanic symptoms, comorbid ADHD or disruptive behavior disorders, and pediatric BD diagnoses.

Female MDEs were characterized by greater clinician-rated emotional lability, excessive weeping, fatigue, appetite disturbances, and parent-reported attention problems. In contrast, male participants displayed more distractibility and conduct problems, as reported by both clinicians and parents. These were the most discriminant sex differences.

Female participants also had more severe—but less discriminant—depressive feelings, low self-esteem, excessive guilt, somatic complaints, internalizing problems, post-traumatic stress, and obsessive-compulsive symptoms (as reported by parents). Male participants, while exhibiting greater motor activity, showed fewer distinguishing internalizing symptoms. Additionally, female participants reported higher levels of anxiety, suicidal ideation, suicide attempts, and NSSI.

### 4.1 Differences in depressive symptoms

Depressive symptoms were more severe in female participants, as assessed by both clinicians using the *CDRS-R* and patients using the *CDI-2*. However, parent-rated depressive symptoms (CDI-2 parent-report module) did not differ between the sexes. Discrepancies between self- and parent-reported measures of depression are well-documented, with self-report generally recommended as the primary source for assessing depressive symptoms in pediatric patients (39). Some studies suggest that nearly half of parents struggle to distinguish between normal mood swings and signs of depression (40), highlighting the challenges in parental recognition of depressive symptoms. However, it must be acknowledged that some authors have questioned the wording of the CDI instrument and the use of discriminative memory required, which can pose difficulties for younger children (41). Given this, we primarily relied on the clinician-rated CDRS-R for our assessment of depression severity. Furthermore, the consistency of results between the CDI-2 self-report module and the CDRS-R strengthens our findings pointing toward a meaningful difference between sexes in both the CDRS-R and the CDI-2 self-report module, while the CDI-2 parent-report module appears less sensitive to detecting these differences.

Higher depressive symptom severity in female participants is in line with previous findings (15) reporting small but significant sex differences in adolescent depression severity and among specific symptoms, including guilt, body image dissatisfaction, concentration problems, depressed mood, fatigue, and health concerns. Our more homogeneous sample, characterized by greater severity and evaluation across a broad range of psychiatric symptoms, allowed us to identify additional differences in affective symptoms, also including mixed features. Additionally, our findings showed greater consistency across raters, and our regression model demonstrated improved fit and classification accuracy.

The higher severity of depressive symptoms in female patients with early-onset mood disorders can be understood within a complex biopsychosocial framework. Several vulnerability factors for depression—such as genetic predispositions, pubertal timing, affective temperament, and cognitive response styles—differ by sex (42). Adolescence brings psychological and physical changes that influence self-perception, interpersonal experiences, and mental health outcomes within a socio-environmental context. Cultural, social, and familial factors are significant, as gender-specific social pressures affect male and female adolescents differently. Girls may also experience a difference in the incidence of adverse childhood events, which we did not investigate, but could partly explain the observed differences (43). Beyond exposure to stressors, the attribution of significance to life events and coping responses differs by sex (44), with relational problems among peers and rumination as a stress-coping strategy more prominent in female subjects (45). This may contribute to the greater expression of core depressive symptoms in female respondents. Recent findings also highlight the role of neurobiological factors in sex-specific susceptibility to depression. Studies of adolescents with depression link gender differences to variations in the hypothalamic–pituitary–adrenal axis response to stress (46). Increased stress reactivity during late puberty and reduced cortisol secretion in early puberty have been identified as risk factors for depression in female adolescents (47). Sex-specific neuroimmune-endocrine factors involved in stress responses and depression have been identified (48), as well as structural nervous system differences observable via magnetic resonance imaging (49). These findings underscore the interplay between biological, psychological, and social factors in explaining the higher depressive symptom severity in female subjects.

Sex differences in appetite disturbances cannot be attributed to differing rates of comorbid eating disorders, which are rare in our sample. This reflects our unit’s referral process, where patients with prominent eating symptoms are directed to a specialized eating disorder unit. Consequently, our homogeneous sample strengthens the study by ensuring that observed differences reflect sex-specific mood disorder expression.

Additionally, clinician- and parent-rated somatic complaints were significantly associated with MDEs in female patients, consistent with previous research (50). We propose that excessive fatigue, appetite disturbances, and somatization in female patients may represent a subset of atypical depressive symptoms. This aligns with studies in adults showing a higher prevalence of atypical MDEs in women (51). However, atypical depression remains understudied in pediatric age.

### 4.2 Differences in mixed symptoms and bipolar disorder diagnosis

To our knowledge, sex differences in subthreshold mixed hypomanic symptoms have not been previously studied in children and adolescents with MDE. Our earlier study assessed fewer adolescents with full-threshold mixed symptoms per the DSM-5 criteria (24). In the present study, mixed features (subthreshold or threshold) were systematically rated using the KMRS in a larger population diagnosed with an MDE. Significant mixed hypomanic features (KMRS total score at threshold) were present in ~10% of the sample and were more frequent in male patients. Male participants were also twice as likely to be diagnosed with pediatric BD at their first psychiatric evaluation for MDE. Prior pediatric studies reported no sex differences in pediatric BD diagnosis (23), but in earlier samples of children diagnosed before age 12, 85% were male patients (52). Consistent with our findings, adult BD cohorts show that female respondents more often experience depressive episodes at onset (53), more depressive episodes throughout illness (54), and mixed features during hypomania or mania (55). Adolescent BD cohorts tend to show hypomanic or manic episodes more frequently in male patients (23). Furthermore, in children and adolescents with severe early-onset mood disorders, conversion from MDD to BD is common during follow-up, affecting up to 45% of cases (56). The DSM-5 criteria limit BD diagnosis at first assessment, as follow-up often reveals additional affective episodes meeting the BD criteria over time. Thus, BD diagnoses at first evaluation in our sample may reflect only a subset of cases where the current MDE is neurobiologically linked to BD. This higher rate of early BD diagnosis in male patients suggests that the DSM-5 criteria more readily recognize BD in male patients, who typically present with manic polarity and distinct episodes, compared to female participants, who more often present with severe MDE and persistent depressive symptoms. Additionally, male patients exhibit more recognizable DSM-5 hypo/manic features in mixed states, as noted in our previous work (24).

Consistent with earlier findings (24), male participants in this study more frequently reached the KMRS threshold, while subthreshold mixed hypomanic features were similarly represented in both sexes, with sex differences in mixed symptom expression. Female participants showed more severe emotional lability, whereas male participants exhibited greater motor activity and distractibility, reflecting a pattern where male patients express more “outer” psychomotor symptoms and female patients display more “inner” emotional lability. While motor activation and distractibility align with DSM-5 mania and mixed features criteria, emotional lability does not. Given the prognostic importance of mixed hypomanic symptoms in adolescents with MDE, even when subthreshold (57), we stress the need for high clinical sensitivity in assessing such symptoms in female patients. Future research should include longitudinal studies to verify these findings and explore the utility of alternative tools, such as the Koukopoulos Mixed Depression Rating Scale (58), which may better capture “inner excitation” symptoms and enhance diagnostic sensitivity beyond current DSM-5 criteria.

We observed that the first psychiatric symptoms emerged over a year earlier in boys than in girls. This aligns with the higher incidence of comorbid ADHD in male participants and is consistent with previous findings on psychiatric comorbidities in mood disorders and the well-documented BD-ADHD comorbidity in pediatric populations (27, 37). These findings highlight the importance of assessing potential neurodevelopmental disorder traits in patients with early-onset mood disorders.

### 4.3 Differences in suicidal ideation and behavior

Our findings confirm the higher prevalence of suicidal ideation and attempts in female patients compared to male patients, consistent with extant literature (15, 24, 59). This aligns with the “gender paradox” in suicidal behavior, where women are more likely to attempt suicide, while men have higher rates of suicide completion and often use more lethal methods (59). Explanations for this phenomenon often point to greater impulsivity and higher rates of substance abuse in men (60).

A recent review of adolescents highlights this differential pattern, identifying both common and sex-specific risk factors for suicide attempts. Female-specific risk factors include eating disorders, PTSD, BD, dating violence victimization, depressive symptoms, interpersonal problems, and previous abortion. Male-specific factors include disruptive behavior, hopelessness, parental separation/divorce, peer suicide, and access to means (61). Similarly, a cohort of Chinese college students found suicide-related outcomes linked to depressive symptoms in female students and anger in male students (62). In our study, suicidal ideation and attempts were more frequent in female participants and correlated with their higher burden of depression severity, further supporting the interplay between depression and suicidal behavior in female subjects.

### 4.4 Differences in externalizing and internalizing comorbidities

Analysis of parent-reported symptoms in the CBCL revealed notable sex differences: female subjects scored higher on internalizing symptoms, while male subjects scored higher on conduct problems. This aligns with the findings in adults with unipolar depression (6) and BD (63), where women are reported to experience comorbid anxiety and post-traumatic symptoms more frequently than men, while men show higher rates of conduct and substance use disorders. Researchers have noted that female participants are more likely to manage emotional problems with internalizing strategies, whereas male participants often adopt externalizing “escaping behaviors” (64).

Of note, no significant sex difference in substance use was observed in our sample, likely due to the study’s focus on early-phase juvenile-onset mood disorders. Such differences may become evident in adults during more chronic phases of these disorders.

Finally, female respondents reported higher levels of self- and parent-rated anxiety symptoms than male respondents, consistent with previous studies in adults (65) and adolescents (66).

### 4.5 Strengths and limitations

This study enriches the literature by providing a detailed, multidimensional analysis of sex differences in severe early-onset mood disorders, a topic rarely explored in comparable populations. A key strength is the use of a single-center sample of referred early-onset mood disorders, ensuring population homogeneity and consistent structured assessments for all participants. However, several limitations should be noted. First, the high severity of depressive episodes in this clinically referred sample may limit the generalizability of the findings to broader clinical populations. Second, while the CDRS-R and KMRS are internationally recognized tools widely used in Italy (24, 27, 67), their validity and reliability in Italian adolescents have not been formally addressed. Reliability analysis in this sample showed acceptable internal consistencies (Cronbach’s alpha: 0.669 for KMRS, 0.796 for CDRS-R). The lower KMRS consistency may reflect the uneven distribution of hypo/manic symptoms, as significant symptoms were confined to participants with subthreshold mixed features, with many participants with a low score of all KMRS items. Third, the study did not assess adverse childhood events, which may vary by sex and influence the findings.

### 4.6 Conclusion

Our study identifies a moderately distinct pattern of sex differences in depressive symptoms among children and adolescents with early-onset mood disorders, partially mirroring adult findings. Consistent with adult literature and limited adolescent studies, we confirm that female participants with MDEs experience more severe depressive symptoms, anxiety, and suicidal behaviors, while male participants more frequently exhibit disruptive behaviors. Moreover, our study highlights sex-based differences in mixed hypomanic symptoms, pediatric BD diagnoses, and early childhood clinical features preceding mood disorder onset.

These findings should be understood within a framework where the interplay of biological, psychological, and social factors begins in childhood and continues into adolescence and adulthood. This interaction shapes sex-specific depression development and intersects with diagnostic systems that often overlook sex and gender differences in key clinical presentations. These observations highlight the importance of integrating a sex- and gender-informed perspective in research, diagnosis, and treatment to enhance outcomes for adolescents with mood disorders.

## Data Availability

The datasets presented in this article are not publicly available because of the nature of the research, due to ethical reasons. Further inquiries can be directed to the corresponding author. Requests to access the datasets should be directed to giulia.serra@opbg.net.
